# Self-Recalibrating Surface EMG Pattern Recognition for Neuroprosthesis Control Based on Convolutional Neural Network

**DOI:** 10.3389/fnins.2017.00379

**Published:** 2017-07-11

**Authors:** Xiaolong Zhai, Beth Jelfs, Rosa H. M. Chan, Chung Tin

**Affiliations:** ^1^Department of Mechanical and Biomedical Engineering, City University of Hong Kong Hong Kong, Hong Kong; ^2^Department of Electronic Engineering, City University of Hong Kong Hong Kong, Hong Kong; ^3^Centre for Biosystems, Neuroscience, and Nanotechnology, City University of Hong Kong Hong Kong, Hong Kong; ^4^Centre for Robotics and Automation, City University of Hong Kong Hong Kong, Hong Kong

**Keywords:** myoelectric control, non-stationary EMG, classification, hand gesture, pattern recognition, convolutional neural network

## Abstract

Hand movement classification based on surface electromyography (sEMG) pattern recognition is a promising approach for upper limb neuroprosthetic control. However, maintaining day-to-day performance is challenged by the non-stationary nature of sEMG in real-life operation. In this study, we propose a self-recalibrating classifier that can be automatically updated to maintain a stable performance over time without the need for user retraining. Our classifier is based on convolutional neural network (CNN) using short latency dimension-reduced sEMG spectrograms as inputs. The pretrained classifier is recalibrated routinely using a corrected version of the prediction results from recent testing sessions. Our proposed system was evaluated with the NinaPro database comprising of hand movement data of 40 intact and 11 amputee subjects. Our system was able to achieve ~10.18% (intact, 50 movement types) and ~2.99% (amputee, 10 movement types) increase in classification accuracy averaged over five testing sessions with respect to the unrecalibrated classifier. When compared with a support vector machine (SVM) classifier, our CNN-based system consistently showed higher absolute performance and larger improvement as well as more efficient training. These results suggest that the proposed system can be a useful tool to facilitate long-term adoption of prosthetics for amputees in real-life applications.

## Introduction

Surface electromyography (sEMG) has become a useful source of control signals for modern prosthetics due to its ease of use and non-invasiveness (Hargrove et al., 2007; Castellini and van der Smagt, 2009). Pattern recognition of sEMG has become a promising techniques for controlling upper limb prosthetics (Scheme and Englehart, 2011). A variety of sEMG features, including time domain and frequency domain features, have been extensively investigated for movement classification with various degrees of success (Hudgins et al., 1993; Zardoshti-Kermani et al., 1995; Phinyomark et al., 2012). Choice of optimal classifiers has also been extensively researched in the past decade, with support vector machines (SVM; Ameri et al., 2014) and linear discriminant analysis (LDA, Chu et al., 2007; Linderman et al., 2009; Phinyomark et al., 2013) having emerged as the common choice for sEMG-based movement classification.

However, sEMG is non-stationary and sensitive to many factors, such as electrode placement, signal crosstalk and recording environment (Scheme and Englehart, 2011). Variation in sEMG can be significant even on a day-by-day basis for the same subject. Hence, performance of the classifiers, and thus the prosthetics, would degrade if they are not recalibrated. This degradation may be minor in a well-controlled laboratory setting but could become a serious problem in real-life clinical applications. This discourages long-term use of neuroprosthetics in amputees. Supervised recalibration of the classifier by asking the user to repeat a strict training protocol daily is possible but would become inconvenient when the number of movement types become large. With even a few minutes of active retraining every day it would become a burden to the user. Alternatively, a self-recalibrating classifier is an adaptive system which can adapt using only the estimated user's intent is desirable since it eliminates the burden of such retraining procedures. A number of adaptive approaches have been applied to enhance robustness of sEMG classifiers (Sensinger et al., 2009; Scheme and Englehart, 2011; Chen et al., 2013; Amsuss et al., 2014; Liu et al., 2016b; Vidovic et al., 2016). Sensinger et al. (2009) proposed several adaptive approaches to expand the training dataset by including some of the online data together with their predictions. These additional data needs to be carefully selected or the performance of the classifier could in fact degrade. It remains an open question for getting the best adaptive paradigm to achieve this. Amsuss et al. (2014) took a post-processing approach to modify the decisions of the LDA classifier by an artificial neural network (ANN) to improve the accuracy by taking into account the history of predictions. However, the classifier system remains unchanged throughout and no new information about changes of sEMG patterns was incorporated. On the other hand, work in (Chen et al., 2013; Liu et al., 2016a; Vidovic et al., 2016) used an adapting LDA approach to compensate for the non-stationarity in sEMG. The pre-trained classifier(s) was adapted using either a new short labeled dataset collected daily (Liu et al., 2016a; Vidovic et al., 2016) or the prediction results directly from the previous sessions (Chen et al., 2013). They demonstrated improved accuracy over a non-adapting classifier but they required daily training to obtain the new labeled data or they used the prediction results directly which may include data that was incorrectly classified. In this study, we aim to develop an adaptive classification system that can compensate for highly non-stationary sEMG without daily retraining.

Convolutional neural network (CNN), proposed by LeCun et al. (1998), has emerged as one of the most powerful machine learning approaches in recent years. The neural network called LeNet-5 was first introduced to classify handwritten and machine-printed characters. Furthermore, implementing CNN using graphics processing unit (GPU) makes it a powerful pattern recognition tool with high efficiency by taking advantages of its parallel computing capability. CNN has demonstrated great success in the areas of image recognition (Krizhevsky et al., 2012), audio classification (Hinton et al., 2012a) and semantic identification (Shelhamer et al., 2017). Recent studies have also shown successful of application of CNNs in the area of biomedical engineering, such as animal behavior classification (Stern et al., 2015), histopathological diagnosis (Litjens et al., 2016), and protein structure prediction (Wang et al., 2016). In this study, we believe that CNN can be a powerful tool in the field of EMG-based hand movement classification as well.

In this paper, we first proposed a CNN based classifier for short latency hand movement classification using sEMG spectrogram as feature. The spectrogram as an input feature was chosen based on our previous work which has shown that when using SVM to classify sEMG the spectrogram feature outperforms that of the previously best feature set (Zhai et al., 2016). Next, we investigated a self-recalibrating CNN classification system which is routinely fine-tuned using prediction results from recent testing session after processed through a label correction mechanism. Testing of our method was performed on the publicly accessible NinaPro database. To validate our results we compared the performance of the proposed classifier with SVM which has been shown to achieve the top performance on the NinaPro database (Atzori et al., 2014; Zhai et al., 2016).

## Materials and methods

The database of the NinaPro project (Atzori et al., 2014) was used in this study. It is a publicly accessible database which has previously been used for research studies on hand movement recognition and decoding (Krasoulis et al., 2015; AbdelMaseeh et al., 2016). The NinaPro Database2 (DB2) contains sEMG data recordings from 40 intact subjects. Each subject is required to perform 49 types of hand movement including 8 isometric and isotonic hand configurations; 9 basic wrist movements; 23 grasping and functional movements and 9 force patterns. Each movement was repeated 6 times with a 3 s rest in between. The 12-channel sEMG signal was sampled at 2,000 Hz and filtered with a Hampel filter to remove 50 Hz power line interference. NinaPro Database 3 (DB3) comprises data of 11 trans-radial amputated subjects with disabilities of the arm, shoulder and hand (DASH) scores ranging from 1.67 to 86.67 (scale 0–100) performing the same 50 hand movements as the intact subjects.

We also tested the classifiers with a smaller number of movement types which could more realistically be implemented on real-world prosthetics. Li et al. (2010) listed 10 types of hand movement which are commonly used in daily life, including wrist flexion and extension, wrist pronation and supination, hand open, and 5 hand-grasp patterns including chuck grip, key grip, power grip, fine pinch grip, and tool grip. We repeated similar testing with these 10 movement types in this study.

**Figure 1:**
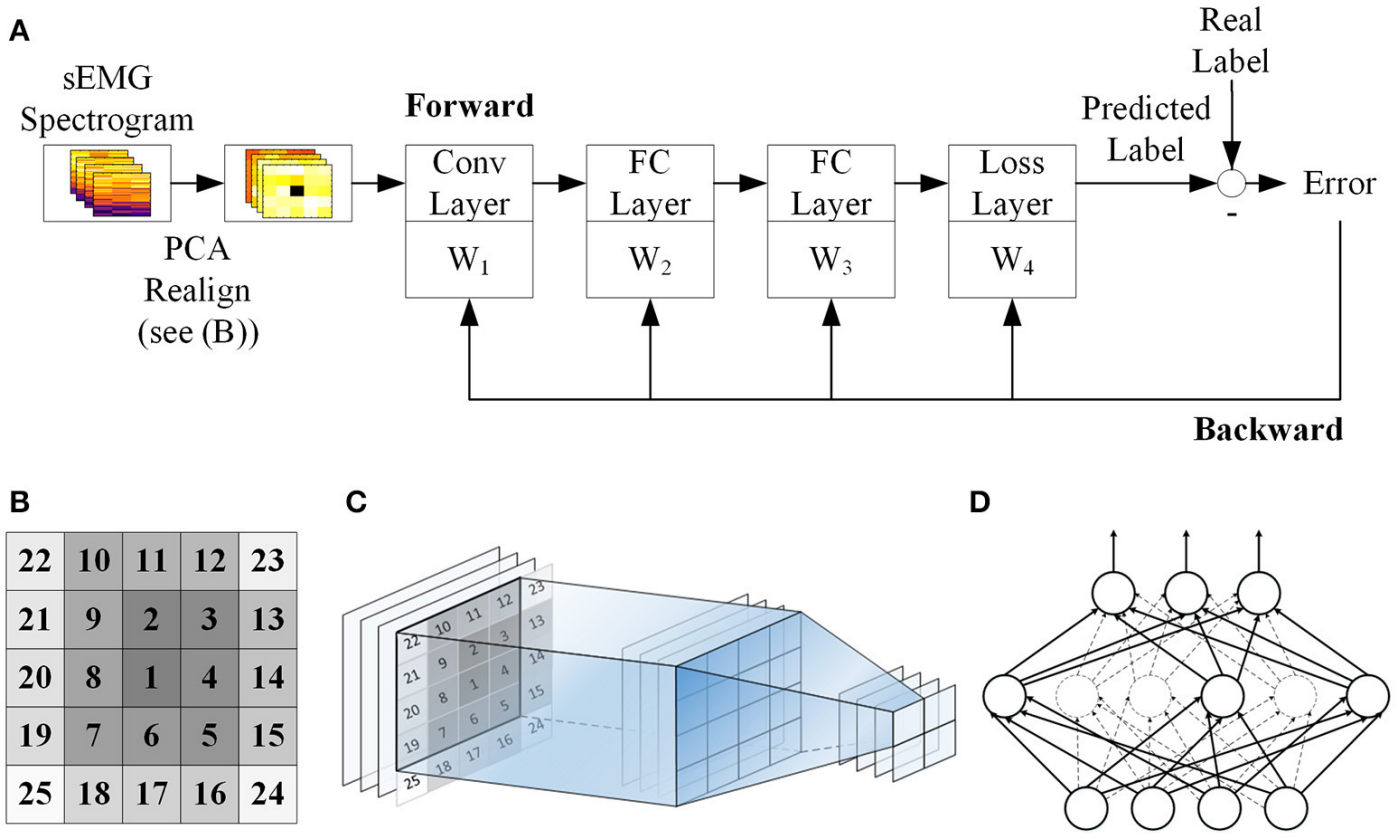
Schematic of the proposed CNN classification. **(A)** sEMG is segmented and spectrogram of each segment is calculated and normalized. Then principal component analysis (PCA) is performed to reduce the dimensionality of the spectrograms before passing them into the CNN classifier. The CNN model contains one convolutional layer (Conv Layer), two full connection layers (FC Layer) with dropout and a softmax loss layer. The network is trained using backpropagation in conjunction with the gradient descent method. **(B)** PCs of sEMG spectrogram are reshaped into a 2D matrix and rearranged in a way such that the most significant PC sits at the center of the matrix while the least significant PCs sit at the corner. The numbers indicate the ranking of the PCs. **(C)** Illustration of the convolutional layer. A 4 × 4 filter is convolved with the 5 × 5 realigned matrix, and gives a resultant 2 × 2 matrix. **(D)** Dropout method. In each training echo, 50% of the neurons in each layer will be randomly picked as dropout neurons and these neurons are ignored in the error propagation and weight update procedures (presented with dashed line).

Figure 1A shows the workflow of the classification scheme in this study. Details of these steps are described in the subsequent sections.

### Data preprocessing

sEMG signals are sectioned into 200 ms (400 samples) segments with 100 ms (200 samples) increments. Delay less than 300 ms is considered acceptable for continuous classification in real-life applications (Englehart and Hudgins, 2003). A prediction of the movement type is given for each segment with each sEMG channel processed independently for spectrogram calculation and normalization.

The spectrogram for each segment of each channel is computed using a 256-point fast Fourier transform (FFT) with a Hamming window and 184-point overlap. Thus, each segment results in a spectrogram calculated at 129 different frequencies (0–1,000 Hz) with 3 time bins. We kept only the first 95 points in frequency of the spectrogram (0–736.54 Hz) because the majority of the sEMG energy was observed within frequency range from 0 to ~700 Hz (Zhai et al., 2016). Hence, the spectrogram of each sample segment results in a matrix of 95 × 3 × 12 (frequency × time bins × channels). The intensity of each spectrogram is then normalized into 0 to 1. For each channel, the 1st and 99th percentiles of the spectral intensity are considered the minimum and maximum value, respectively. Values beyond this range will be forced to 0 or 1. To improve computational efficiency and performance, we vectorize the normalized spectrogram matrices channel by channel and then apply PCA to it. Only the scores of the first 25 principal components (PCs) of each channel are used for the classification, hence, a total of 300 PC scores. We have shown previously that the first 100–500 PCs are sufficient to achieve good classification accuracy (Zhai et al., 2016). As a result, each spectrogram matrix is reduced to a dimension of 25 × 12 (PC × channels) after PCA.

### Classification

Previous studies have shown that SVM with radial basis function (RBF) kernel offered the best classification results for DB2 using sEMG spectrogram as input features (Atzori et al., 2014; Zhai et al., 2016). Hence, SVM is used to benchmark our CNN-based system in this study. An open source C++ library LIBSVM (Chang and Lin, 2011) was used to implement the SVM classifier. The optimal hyper-parameter pair (c, γ) was obtained with a four-fold cross validation (Atzori et al., 2012).

Figure 1A shows a schematic for our CNN classifier. Our CNN model contains 1 convolutional layer (Conv Layer), 2 fully connected layers (FC Layer) with dropout and a softmax loss layer. The softmax loss layer computes the cost function using the normalized exponential function. It also outputs the probabilities of all movement types considered in the current prediction. Each layer is trained by backpropagation. An open source MATLAB toolbox MatConvNet was used to implement the CNN classifier (Vedaldi and Lenc, 2015).

Before inputting into the CNN, the resultant vectors of PC scores are first rearranged in to a 2D matrix such that, for each channel, the 25 × 1 vector becomes a 5 × 5 matrix. In this way, each of the sEMG segments is treated like a 2D image and the 12 channels mimic the RGB channels in a color image. Furthermore, to optimize the use of the CNN, the PCs are rearranged in a way such that the score of the most significant PC sits at the center of the matrix while the least significant PCs sit at the corners (Figure 1B). In this way, the major PCs can be captured by most of the convolving filters and hence maximize their contribution in the network. This rearrangement can provide an additional 1–2% improvement in overall accuracy. Figure 1C shows the forward projection of the convolutional layer using a 4 × 4 filter.

In the FC layers we use rectified linear units (ReLU) as activation function which has been shown to help avoid problem of vanishing gradient (Glorot et al., 2011), and hence effectively speed up training. We also apply dropout method to reduce overfitting (Hinton et al., 2012b). In each training echo, 50% of the neurons in the fully connected layers will be randomly dropped from error propagation and weight update (Figure 1D). Randomly selecting the dropout neurons in this manner should reduce the chances of coadaptation of the parameters and hence, decrease the interdependence of neurons which can lead to overfitting.

### Self-recalibration

**Figure 2:**
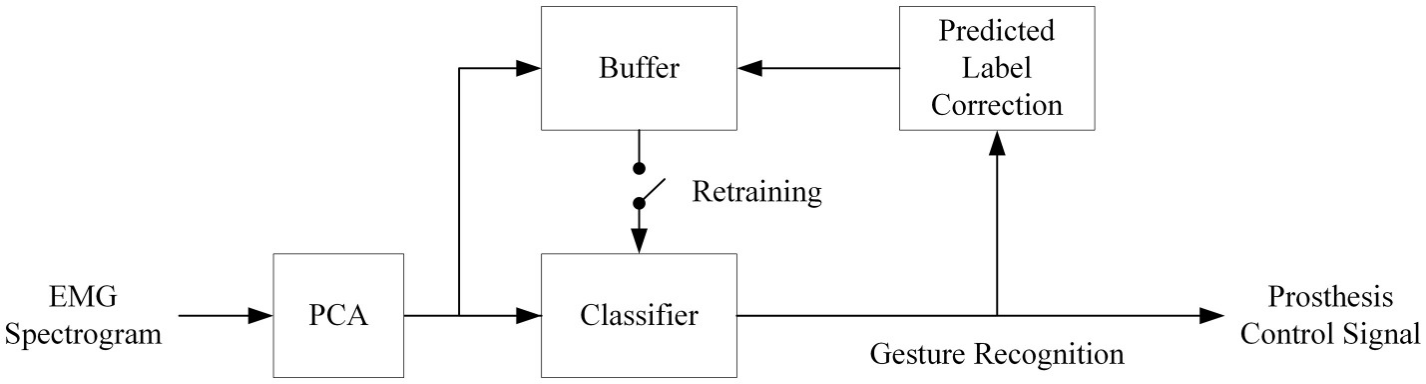
Block diagram of the proposed self-recalibration classifier. Retraining of classifier is executed whenever the buffer has been filled up with the PC score and updated label. The size of the buffer can be flexibly designed to balance performance and computational load as well as for gestures of interests. The retraining follows the same mechanism as shown in Figure 1A.

Self-recalibration of the classifier is critical for real-life prosthetic application due to the day-to-day (and even session-to-session) variability of sEMG. In order to simulate this scenario, the first set of the six repetitions of movements in DB2 and DB3 was selected as the initial training set, while the other five repetitions were tested one by one with the classifiers. The prediction results from previous session are fed back to retrain the classifiers prior to each testing session (Figure 2). To improve performance, the predicted labels are first corrected offline using a multi-vote method. The assumption is that neighboring sEMG segments are likely belonging to the same hand movement type. A similar assumption was used in developing a self-correcting classifier (Amsuss et al., 2014).

Assume *L*^*i*^ denotes the predicted label of the *i*^*th*^ segment from the previous testing session. This label can then be updated based on the label which occurs the most often in the segments in the adjacent ±*x* segments.

(1) $$\begin{array}{l} L^{i}\leftarrow mode\left(L^{i-x},\;L^{i-x+1},\cdots \;,L^{i},\cdots \;,\;L^{i+x\;}\right) \end{array}$$

where x will be picked to optimize the accuracy of the relabeling.

For the CNN, we can also consider an alternative label update to *L*^*i*^ based on the median probability. Let *P*(*i,j*) denotes the predicted probability of the *j*^*th*^ movement class for *i*^*th*^ segment. For each *j*, we compute the median probability, $\tilde{P}\left(i,j\right)$, over the adjacent ±*x* segments,

(2) $$\begin{array}{l} \tilde{P}\left(i,j\right)=median(P\left(i-x,j\right),\;P\left(i-x+1,j\right),\;\ldots ,\\ \;P\left(i,j\right),\ldots ,P(i+x,j)) \end{array}$$

Then we find *j* with the maximum $\tilde{P}\left(i,j\right)$ and use it as the updated label for segment *i*,

(3) $$\begin{array}{l} L^{i}\leftarrow \arg\underset{j}{\max}\left(\tilde{P}\left(i,j\right)\right) \end{array}$$

The median, instead of mean, is used here to minimize the effects of outliers. This updated data is then used to retrain the classifiers.

In the self-recalibrating classifier, the most recent session was fed back to update the classifier. In fact, the amount of feedback data can be flexibly chosen based on performance, computational load, and gestures of interest. We also considered the extreme when results from all previous sessions were kept to update the classifier. The three scenarios to be compared are as follow.

- No recalibration: The classifier is only trained once using the initial training data set.
- All-Session recalibration: The classifier is retrained using the initial training data set plus the prediction results from all the previous testing sessions. This serves as an estimate for maximum expected performance but the continuous accumulation of the data in long run is impractical for real-life application.
- Last-Only recalibration: The classifier is retrained using only the prediction from the most recent testing session.

### Performance evaluation and statistical analysis

The classification accuracy was calculated in a class-specific manner. The accuracy, *Acc*_*i*_, for subject *i* is calculated as,

(4) $$\begin{array}{l} Acc_{i}=\frac{1}{M}\overset{M}{\underset{j=1}{\sum }}{\left[\frac{\#\;correct\;segments}{\#total\;segments}\right]}_{j} \end{array}$$

where M is the total number of movement types. The class-specific accuracy is suggested to be a preferred metric over global accuracy for quantifying the performance of the classifier (Ortiz-Catalan et al., 2015). In fact, we have also balanced the number of trials for all the movement types (including rest) in this study which minimizes the bias in calculating the accuracy.

All pairwise comparisons were based on one-way ANOVA with repeated measures followed by Bonferroni *post-hoc* analysis. Significant level was set at *p* < 0.05. Unless specified otherwise, all results are presented as mean ± 1 standard error.

## Results

### Evaluation of CNN structure and recalibration mechanism

**Figure 3:**
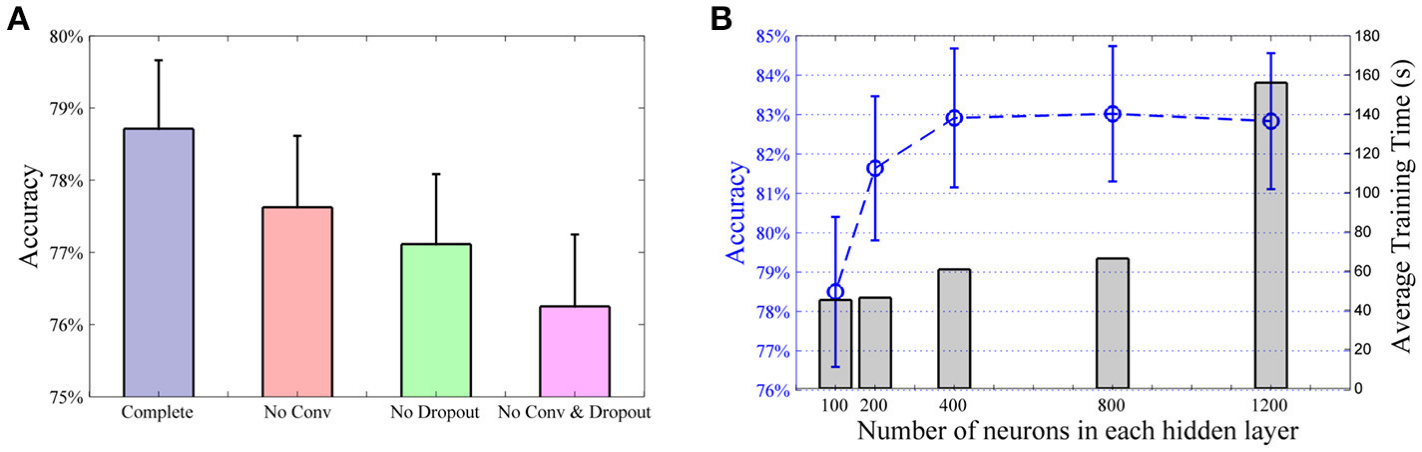
Analysis of CNN structure. **(A)** Effects of convolutional layer and dropout method. Compared to the model with no convolutional layer nor dropout layer (i.e., a standard ANN), incorporating the convolutional layer or the dropout resulted in a rise in classification accuracy of 1.4% and 0.9%, respectively. Furthermore, the complete CNN classifier offered a 2.5% improvement than the standard ANN. All pairwise comparisons are statistically significant. **(B)** Average accuracy (blue line) and training time (gray bars) of the first 10 subjects with different number of neurons in CNN hidden layers. Same number of neurons are used in each hidden layer.

To optimize the design of the classifier system, we performed a series of simulations using two third of the movement repetitions, same as the NinaPro paper (Atzori et al., 2014), to train the classifiers with data from the first 10 subjects of DB2. While it is difficult to obtain a globally optimal network structure, these results provide some guidance to select a good network design that balance between performance and computational cost. First, we consider the effects of the convolutional layer and dropout layers of the CNN classifier (Figures 1B–D show the major components of the CNN classifier). Figure 3A shows a comparison of the overall accuracies of the complete CNN classifier and compromised versions without the convolutional and/or the dropout layers. In the models without the convolutional layer, the layer was replaced by a fully connected layer and hence the total number of layers conserved. For the model without neither the convolutional layer nor the dropout layers, it essentially becomes a traditional ANN. The convolutional layer and the dropout together contributed a 2.5% improvement in classification accuracy.

Next we tested the performance of the CNN classifier with different numbers of neurons in the hidden layers. Here we use the same number of neurons in each layer. Having a larger number of neurons improved the performance with the average classification accuracy peaking at around 800 neurons (Figure 3B). Increasing the number of neurons to 1,200 added little or no improvement to the classifier but resulted in a large increase in computational time. In our implementation, the difference in computational cost and accuracy is very small between 400 and 800 neurons. We have used 800 neurons in our network for the rest of the study.

**Figure 4:**
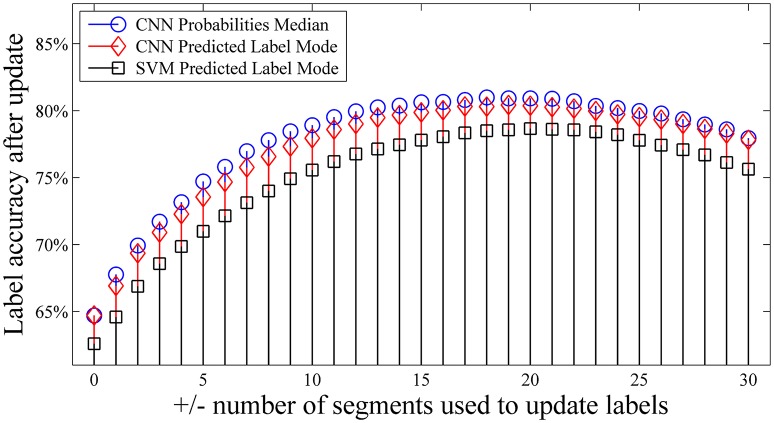
Effects of number of segments used for label updating (Equations 1–3). The data in the first repetition was used to train the classifiers, and was tested with the second repetition. Accuracy was calculated by comparing the updated labels against the ground truth for the first 10 subjects.

Finally, we evaluated the optimal windows size for the label updating mechanisms as described by Equations 1–3. We recomputed the label accuracy after update using different numbers of segments. Figure 4 shows that the accuracy of the updated labels can be increased by as much as ~15% when compared with the ground truth. For both our proposed self-recalibrating CNN classifier and SVM, we used a window of ±10 segments to update the predicted labels which gives a good balance between performance and latency in dealing with the NinaPro database. Figure 4 also shows that label update based on the median probability (Equations 2 and 3) is preferred for our CNN classifier.

### Performance of baseline classifiers

We first tested a “baseline” version of the classifiers. The baseline classifiers were trained in exactly the same way as in the NinaPro study (Atzori et al., 2014). For each movement type, the 1st, 3rd, 4th, and 6th repetitions were used as the training set, while the other two repetitions were used as the testing set. The overall accuracies averaged over all subjects and all movement types, are summarized in Table 1 and Supplementary Table S1. The average accuracy of SVM on all movement types is 77.44%, which is higher than the best results (75.27%) reported in the NinaPro study using Random Forests with a combination of four features (Atzori et al., 2014). The accuracy of the proposed CNN classifier is slightly higher than that of SVM (1.13%). The confusion matrix from the CNN classifier shows that the majority of error was due to misclassifications into movements of the same class (Supplementary Figure S1). The small improvement of CNN over SVM was also observed in testings with intact subjects on the 10 movement subset (88.42% vs. 87.86%) and with amputee subjects (73.31% vs. 72.01%). The improvement was consistent for all subjects tested (Supplementary Figures S2, S3). (Amputee Subject 7 had a very low classification accuracy (<18%) in all testing for both classifiers, probably because his entire forearm has been lost. Hence, Subject 7 was eliminated from all of our analysis.).

**Table 1:** Summary of classification accuracy for baseline classifiers.

	**SVM%**	**CNN%**
	**Intact subjects (*****n*** = **40)**	
** All movement**	77.44	78.71
Basic movement (index 2 to 18)	81.07	82.22
Grasping and functional movement (index 19 to 41)	71.08	72.62
Force pattern (index 42 to 50)	88.56	89.54
	**Intact subjects (*****n*** = **40)**	
** 10 Movement subset**	87.86	88.42
	**Amputees (*****n*** = **10)**	
	72.01	73.31

**Figure 5:**
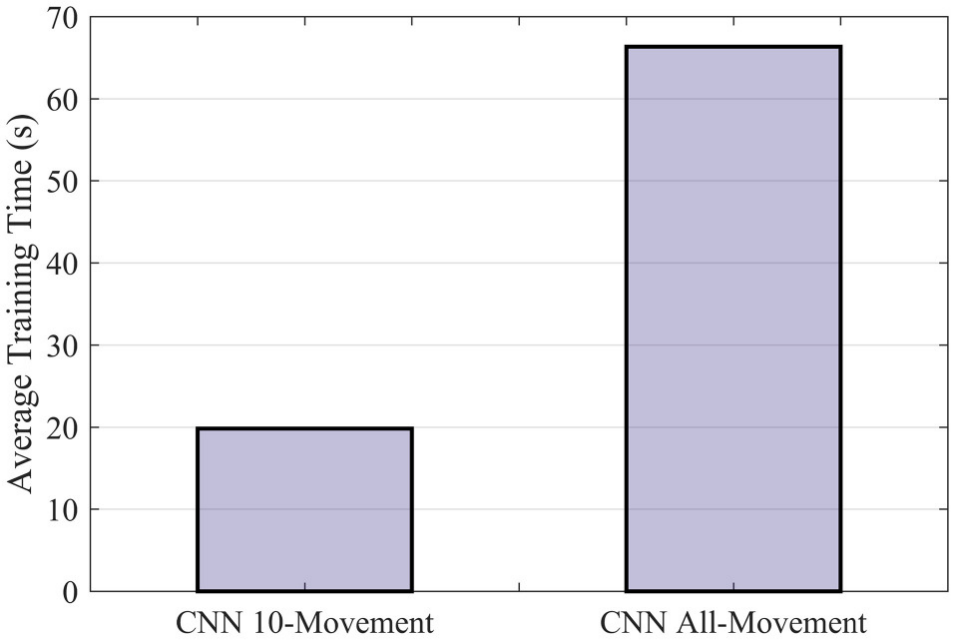
Average training time of CNN for one subject. The CNN model was implemented with NVIDIA CUDA® Deep Neural Network library (cuDNN) to be run on a Nvidia GTX 980M GPU.

Although the difference in classification accuracy is small, computation with CNN could be quite efficient despite the complexity. We implemented the CNN classifier on NVIDIA CUDA® Deep Neural Network library (cuDNN; Chetlur et al., 2014) to be trained on a NVIDIA GTX 980M GPU. It took 19.83 s to train the CNN for one subject on 10 movement subsets and 66.34 s on all 50 movement types (Figure 5). The training of CNN is sufficiently fast to allow recalibration online to compensate for variation in sEMG signals. The results also show that CNN can scale quite efficiently when dealing with more movement types. We also tested SVM using four cores parallel computing with CPU (Intel i5-6600 with 16GB DDR4 RAM). The scalability appeared to be worse for SVM (Supplementary Figure S4, 23.71 s for 10 movement types vs. 561.62 s for 50 movement types). Further optimization for SVM implementation may resolve this issue but few recent works have found available for GPU acceleration of SVM (e.g., Athanasopoulos et al., 2011).

### Performance of self-recalibrating classifiers

We then investigate a self-recalibrating system based on these two classifiers. We would like to emphasize that after the initial training, no new data with true labels were provided to the classifiers. Instead, the classifiers were retrained based on only the predictions from previous sessions.

#### Intact subjects (DB2)

**Figure 6:**
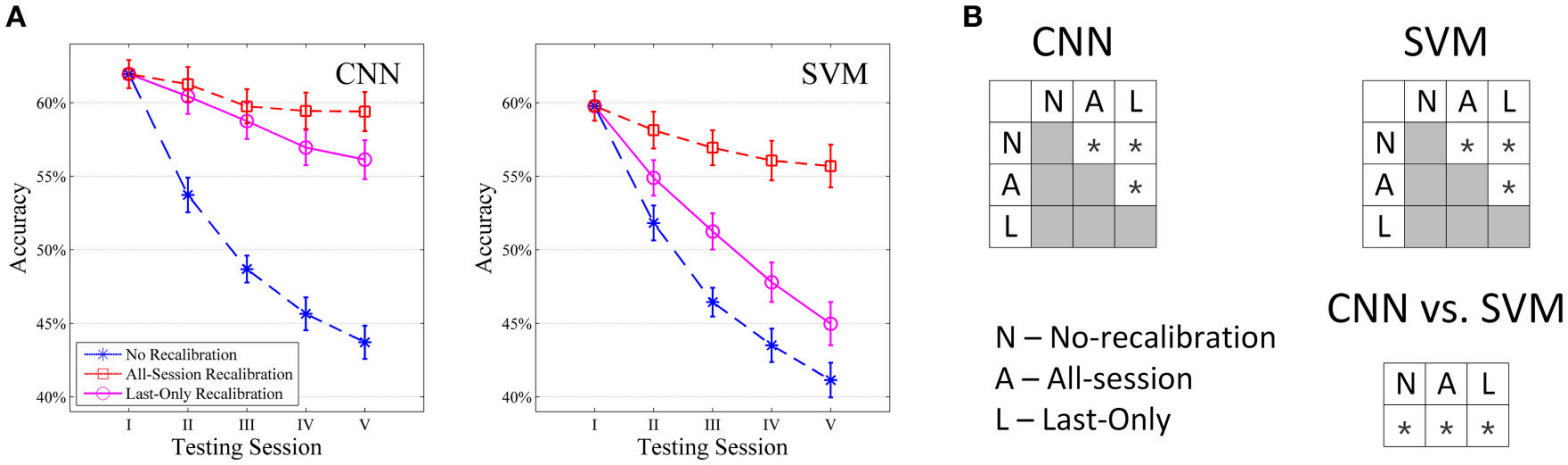
Comparison of CNN and SVM in intact subjects (*n* = 40) tested with all movement types. **(A)** Average session-to-session accuracy in different self-recalibration scenario. Repetition 1 of movement was used as the training data, and repetitions 2 to 6 were tested one by one with or without recalibration. **(B)** Statistical analysis of session-to-session performance. We compare session-to-session difference among the three scenarios, as well as between CNN and SVM. ^*^ Indicates pairwise statistically significant difference (*p* < 0.05).

The session-to-session performance of both our CNN classifier and SVM for intact subjects are shown in Figure 6A. For each simulation, only the first repetition was used as training data. The first testing session was then performed on repetition 2 (Session I), after which the predicted labels were updated according to Equations 1–3 and the classifiers recalibrated using these updated labels. The same procedure was then repeated for repetitions 3, 4, 5, and then 6 (Session II to V). Each recalibration took 21.78 s for CNN when considering all 50 movement types (5 s each). When no recalibration was performed, the accuracies of both classifiers dropped monotonically session by session. This reflects a pretty rapid drift in sEMG pattern from repetition to repetition in the NinaPro dataset such that at the fifth testing session, a significant drop in performance has been accumulated for both CNN (18.66%) and SVM (19.19%), although CNN consistently offered higher accuracy than SVM for all testing sessions. This drop in performance is not due to specific choice of sEMG features *per-se*. We have tested a number of commonly used sEMG features on the classifier (e.g., RMS, Autoregressive Coefficient, Mean Frequency, Median Frequency, Frequency Ratio, Peak Frequency) and a similar drop in performance with even lower accuracies was observed in all of them (data not shown).

All-Session recalibration offers large improvement in performance and robustness for both classifiers, which gives an estimate of maximum improvement we could expect from such self-recalibrating system. The accuracy dropped by only 2.63% for CNN and 4.33% for SVM by the fifth testing session, which corresponds to an average of 12.08% and 11.11% improvement from the unrecalibrated classifiers, respectively (Figure 6A). Not only that CNN offers a larger improvement, the absolute average accuracy of CNN is also higher than that of SVM (Figure 6B).

**Figure 7:**
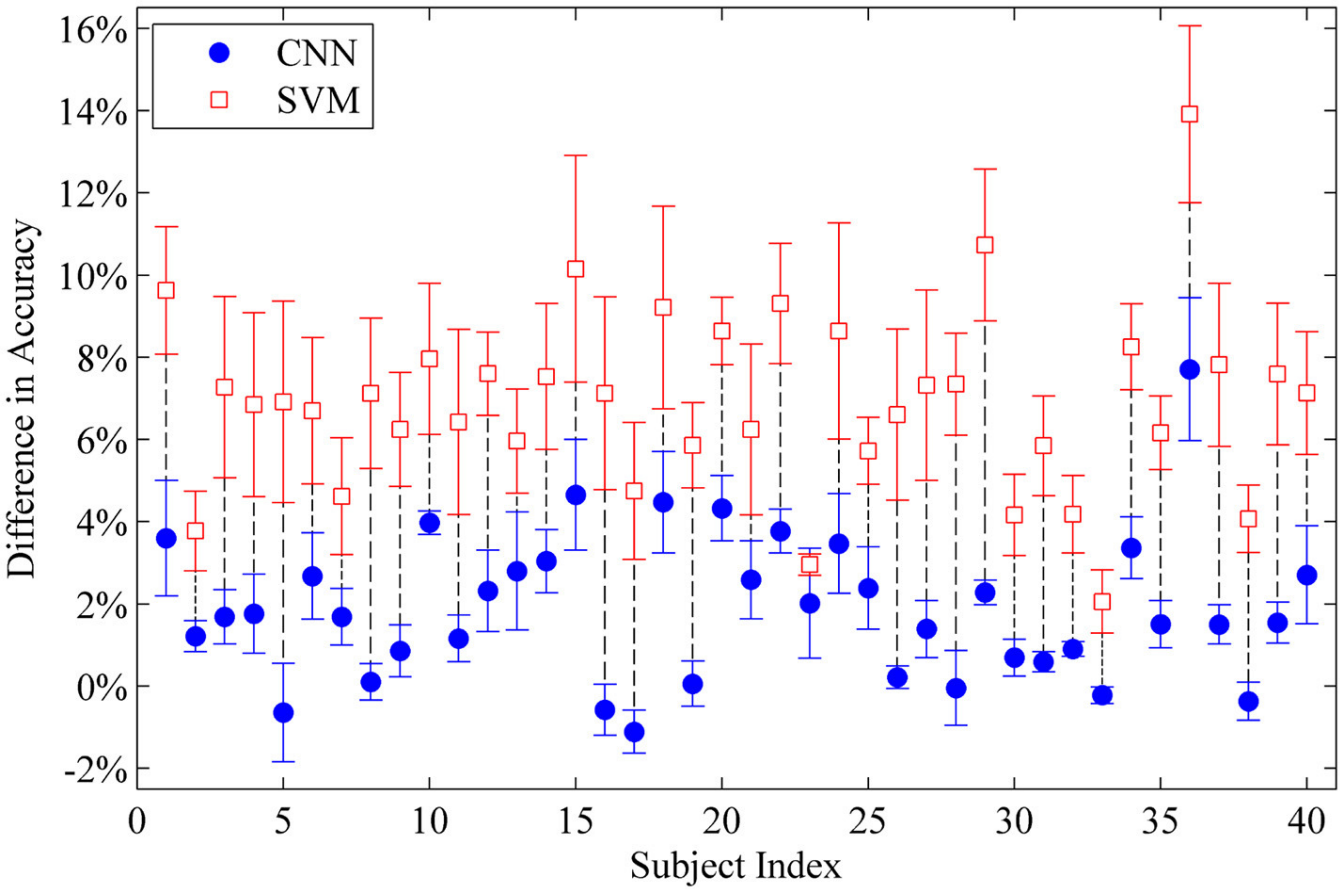
Difference in classification accuracy between All-Session and Last-Only recalibration for each intact subject tested with all movement types. Each point represents the average difference over sessions II to V. The median difference for CNN and SVM is 1.68% and 6.92% respectively.

Last-Only recalibration method, which is more practical for real life application, offers comparable improvement for the CNN classifier to the All-session recalibration approaches, but much smaller improvement for SVM (10.18% for CNN vs. 4.20% for SVM averaged over 5 testing sessions) (Figure 6 and Table 2). Furthermore, Figure 7 shows the difference in classification accuracy between All-Session and Last-Only recalibration for each subject. The difference is only 1.68% (median) for CNN while that for SVM is 6.92%. The trend is consistent for each of the 40 subjects tested.

**Table 2:** Difference in classification accuracy of the self-recalibrating systems from the No-recalibrating case.

	**Session**	**Session**	**Session**	**Session**	
	**II**	**III**	**IV**	**V**	**Average**
Intact—All Movement (Figure 6)					
CNN	6.41%	9.95%	11.47%	12.88%	10.18%
SVM	2.94%	4.68%	4.58%	4.59%	4.20%
Intact—10 Movement (Supplementary Figure S6)					
CNN	3.33%	6.80%	7.84%	9.92%	6.97%
SVM	2.11%	3.56%	3.58%	3.45%	3.18%
Amputee—10 Movement (Figure 8)					
CNN	2.37%	3.52%	3.31%	2.76%	2.99%
SVM	1.33%	−1.13%	−3.29%	−2.86%	−1.49%

*Positive value indicates higher accuracy than the No-recalibrating case*.

While the absolute accuracies appear relatively low in the results shown above, we would like to emphasize that we have only used a single repetition as the initial training set. By using the first 3 repetitions for initial training, the absolute performance can be readily improved by ~10% for all testing sessions (Supplementary Figure S5). It also shows that with Last-Only recalibration, the performance of SVM was even worse than the case with no recalibration at all, suggesting that the SVM-based system is more sensitive to variation of the data over different sessions. Data with more repetitions as training set should further improve the performance, but it would also increase the burden of sEMG collection. Further studies could identify the appropriate balance between these two.

Testing on 10 movement subset showed higher overall accuracy (by ~13%) with similar trend as in testings with all movement types (Supplementary Figure S6), with an average improvement in accuracy of 6.97% and 3.18% for CNN and SVM, respectively (Table 2). Despite a smaller difference in performance, Last-Only recalibration of CNN is still much better than that of SVM.

#### Amputee subjects (DB3)

**Figure 8:**
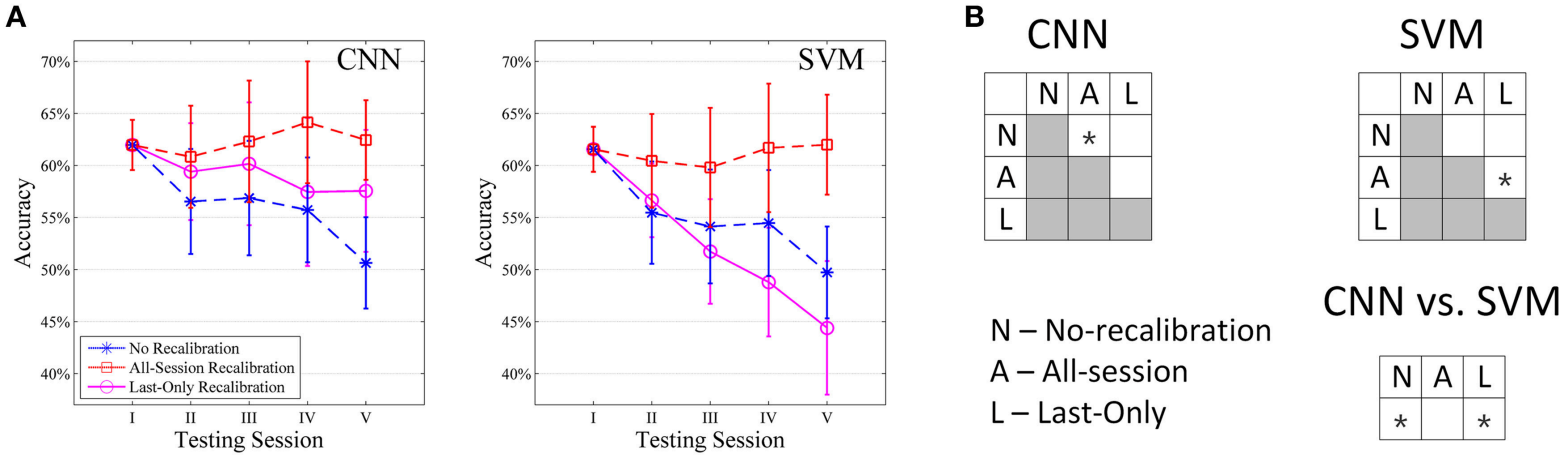
Comparison of CNN and SVM in amputee subjects with myoelectric prostheses experience (*n* = 5) tested with 10 movement subset. **(A)** Average session-to-session accuracy in different self-recalibration scenario. Repetition 1 of movement was used as the training data, and repetitions 2 to 6 were tested one by one with or without recalibration. **(B)** Statistical analysis of session-to-session performance. We compare session-to-session difference among the three scenarios, as well as between CNN and SVM. ^*^ indicates pairwise statistically significant difference (*p* < 0.05).

We have also tested the recalibrating performance of our CNN classifier on the amputee subjects in NinaPro Database 3. We tested the performance only on amputee subjects with experience in myoelectric prostheses (4–13 years). For testing on 10 movement subset, a similar trend as in intact subjects is observed although the accuracy is generally lower (Figure 8A). The recalibrated CNN classifiers generally perform better than unrecalibrated ones (+2.99% on average, Table 2), although statistical significance is weaker in amputees, primarily due to larger variability in these subjects and smaller sample size (Figure 8B). It is worth noting that the average performance of Last-Only recalibrated SVM is even lower than the unrecalibrated SVM (−1.49% on average, Table 2) suggesting that SVM is more sensitive to nature of the data over different sessions. We have also repeated the simulations on all amputee subjects and amputee subjects with remaining forearm >70% and similar trends could be seen for these cases (Supplementary Figure S7). Testing of amputee subjects on all movement types is unrealistic particularly when data from a single repetition is used for initial training. Despite a similar trend as in other testings, this resulted in a low accuracy in first testing session (~40%) which would not be useful for any meaningful recalibration.

## Discussion

We have proposed a CNN-based framework for hand movement classification based on dimension-reduced sEMG spectrograms. By combining a CNN classifier with a simple label updating mechanism, the classifier provides an effective self-recalibration capability to maintain a robust session-to-session performance for both intact and amputee subjects. In our simulations, we showed that the self-recalibrating CNN classifier can offer an average of 10.18% increase in accuracy when compared to the unrecalibrated classifier, while the SVM-based system showed only 4.20% increase in accuracy. The label correction mechanism has been effective in maximize the use of the prediction data such that the performance could be maintained even though the accuracy only started at 61.7% (Figure 6). All subjects showed improved performance with recalibrated CNN but several subjects showed poorer performance using Last-Only recalibrated SVM. These results support that our CNN framework could be a useful tool to compensate for continuous drift in sEMG signals without routine retraining. To adopt this self-recalibrating system for day-to-day application of neuroprosthetics, the classifier could be updated in the background with the same mechanism for a suitable time interval (e.g., every 1 h as one session) without the need of active retraining by the user. Future study will investigate the performance of our proposed system for long-term use.

The convolutional and the dropout layer of CNN provide certain degree of regularization and the use of ReLU activation function also helps speed up training and avoid the need for pre-training. It is also intuitive to incorporate new data to update the neural network that partially retains the memory of the information from previous data and provides a desirable initial condition for fine-tuning the network using new testing data. The popularity of CNN and other deep learning frameworks in image processing, speech recognition and so on have led to more efficient computational tools which have essentially improved the speed of the training process and eased the complication of implementation. For instance, we have used the NVIDIA CUDA® Deep Neural Network library (cuDNN; Chetlur et al., 2014) to speed up training of our CNN classifier. Our CNN classifier can be effectively parallelized with GPU such that the training speed was faster than SVM. In fact, the overhead of incorporating more movement types is much less on CNN than SVM (Figure 5 and Supplementary Figure S4). These advantages make the CNN a more flexible platform for controlling more powerful neuroprosthetics.

Two recent papers have also adopted CNN for sEMG hand movement classification. Atzori et al. (2016) applied a CNN classifier on the NinaPro dataset, which reached an average accuracy of 60.27% on DB2 taking a total training time of 1 h and 42 min. However, the performance was lower than that of the best classical classification methods (Random Forests with all features, 75.27% (Atzori et al., 2014). In this paper, we have showed that our design offers a much higher performance (78.71%, Table 1) and faster training time (~44 min for 40 subjects) even on a less powerful GPU (NVIDIA GTX 980M vs. NVIDIA Titan-X GPU). Geng et al. (2016) employed an image-classification framework with CNN to show that instantaneous sEMG signal obtained from high density sEMG recording (128 channels) can be a useful feature for hand movement classification. The idea of instantaneous sEMG image is attractive for neuroprosthetic application with minimum delay but it will also require more resources to handle the high density inputs. The advantage of low latency was not enjoyed by the low density NinaPro dataset because the classification accuracy for all 52 movement types on DB1 using a short 10 ms windows was only ~65% as shown in their work. The higher computational load will be a drawback on a recalibrating system as addressed in this study.

We used SVM as the benchmarking classifier in our study since it previously offered the best performance for NinaPro database using the sEMG spectrogram (Zhai et al., 2016). On the other hand, a number of recent studies on self-recalibrating hand movement classifiers have been based on LDA (Chen et al., 2013; Amsuss et al., 2014; Vidovic et al., 2016), which is a simple and easy to implement algorithm. However, performance of LDA on the NinaPro database has been shown to be lower than other competing classifiers (Atzori et al., 2014) and hence it was not used in our study (our preliminary testings showed that performance of LDA was ~10% lower than SVM and CNN). This may be because the number of movement types is large and the sEMG properties drift quickly from session to session in this database, which make it difficult to estimate the probability distributions for each class reliably and hence fuzzy linear boundaries. Nevertheless, publicly accessible databases like NinaPro are still a valuable resource which allow direct comparison of different algorithms.

Several aspects of performance evaluation could be more thoroughly investigated in future studies. First, online experiment will be required to fully validate our self-recalibrating system as offline and online performance may not always correlate. In this study, we have performed the self-recalibration testing according to the sequence as the subject performing movement during the experiment. This has preserved the temporal profile of sEMG to some extend which mimics an online experiment. We have also used class-specific accuracy which is suggested to be a less biased metric for performance evaluation (Ortiz-Catalan et al., 2015). As such, we believe that our offline analysis is still a valid reference for online performance. Second, during real-life conditions people rarely hold sustained constant force contractions as are presented in the NinaPro database. Hence, a more extensive dataset over multiple days with more realistic movement will grant more thorough evaluation of our system in terms of both design of the network and the recalibration mechanism.

## Author contributions

XZ and CT designed the research. XZ performed the simulation and analyzed the results. BJ, RC, and CT reviewed and revised the analysis. XZ and CT wrote the main text of the manuscript. All authors reviewed, revised, and finalized the manuscript.

### Conflict of interest statement

The authors declare that the research was conducted in the absence of any commercial or financial relationships that could be construed as a potential conflict of interest.
