# Supplementary material for: Self-Recalibrating Surface EMG Pattern Recognition for Neuroprosthesis Control Based on Convolutional Neural Network
Source: Front Neurosci. 2017 Jul 11;11:379. doi: 10.3389/fnins.2017.00379 (PMC5504564; doi:10.3389/fnins.2017.00379)
Supplement: Supplementary file 1 [file DataSheet1.pdf]

Supplementary Material

Self-Recalibrating Surface EMG Pattern Recognition for Neuroprosthesis Control based on Convolutional Neural Network

Xiaolong Zhai, Beth Jelfs, Rosa H. M. Chan, Chung Tin*

*** Correspondence:** Chung Tin, PhD.: chungtin@cityu.edu.hk

1 Supplementary Figures

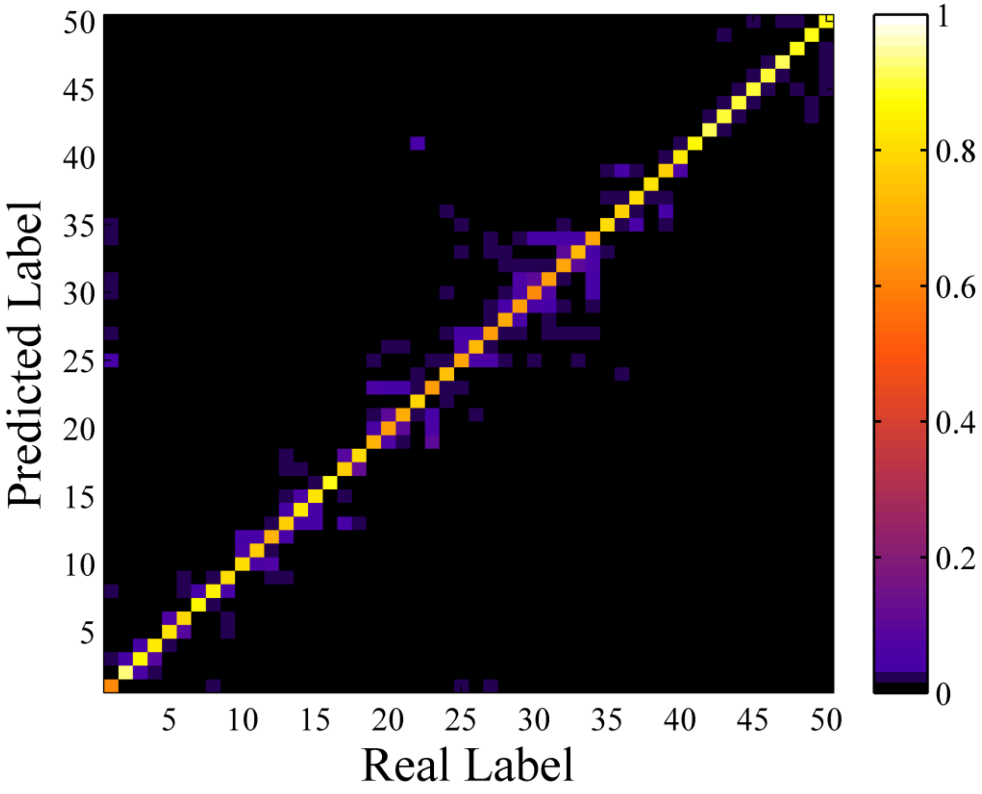

Supplementary Figure S1. Confusion matrix of prediction using CNN for all 50 movements. Most of the confusion occurs with movements that have indices close to the index of the actual movement. In other words, the confusion mainly occurs in the same class of movement.

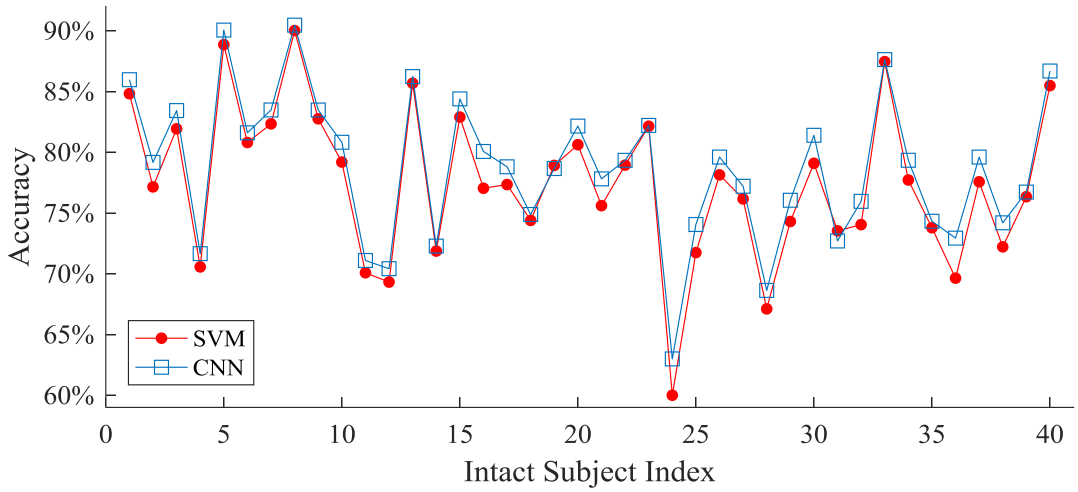

Supplementary Figure S2. Comparison of CNN and SVM subject-to-subject accuracy of baseline classifier in intact subject with all movement types.

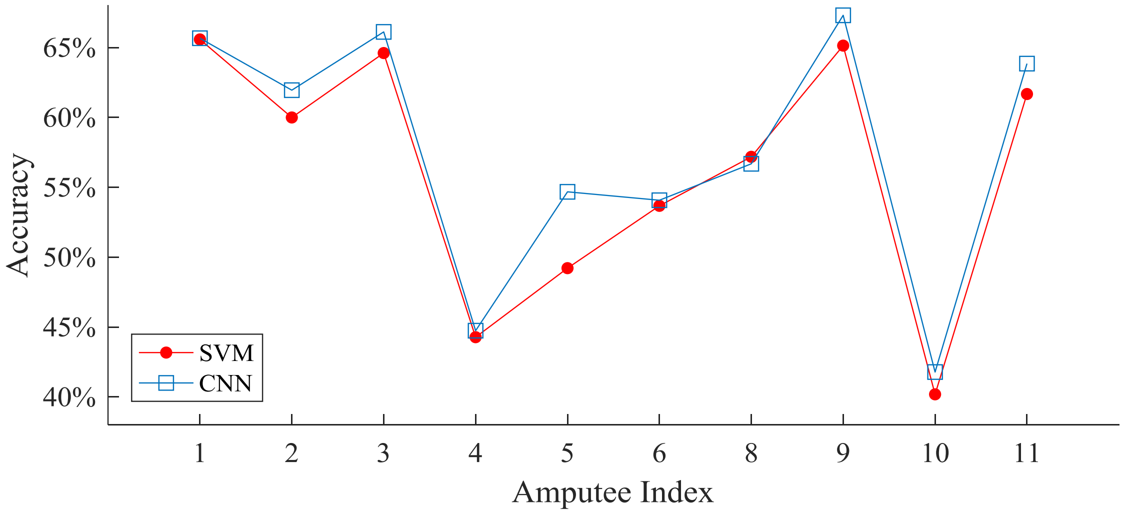

Supplementary Figure S3. Comparison of CNN and SVM subject-to-subject accuracy of baseline classifier in amputee subject with all movement types.

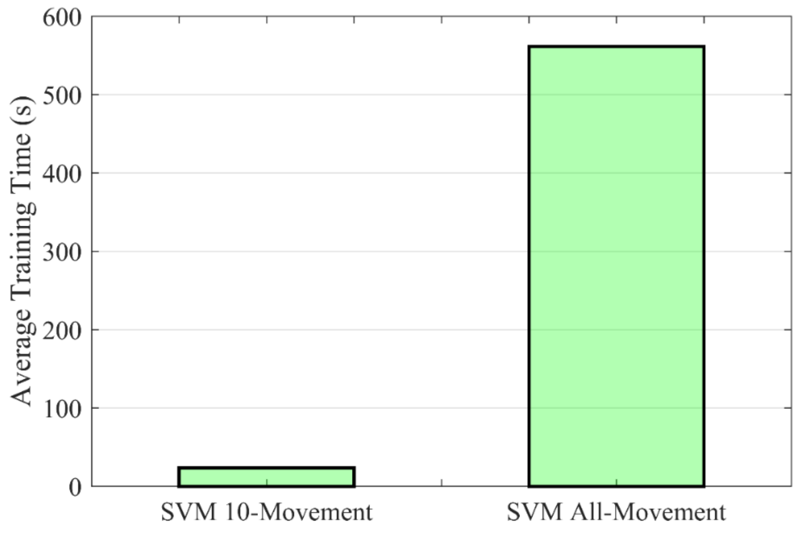

Supplementary Figure S4. Average training time of SVM for one subject. The training procedure was repeated 10 times to obtain an average training time. The SVM model was implemented for 4-Core parallel computing to be run on an Intel i5-6000.

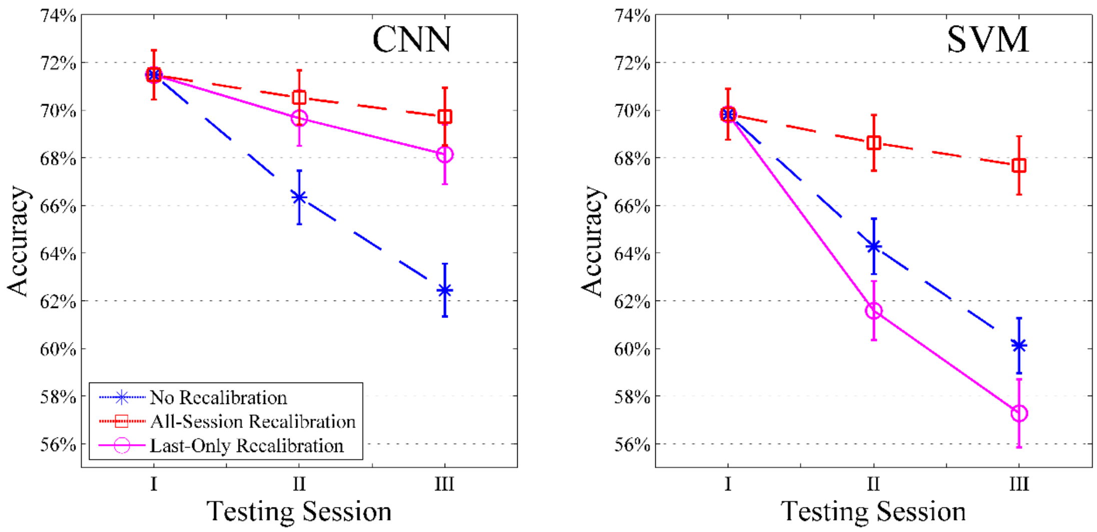

Supplementary Figure S5. Average session-to-session accuracy in self re-calibration scenario of intact subject using 3 repetitions for initial training (n=40) tested with all movement types. Repetitions 1, 2 and 3 of movement were used as the training data, and the other 3 repetitions were tested one by one with or without recalibration. The overall performance of CNN and SVM were increased by about 10% when compared with results in Fig. 6a which used only one repetition for initial training.

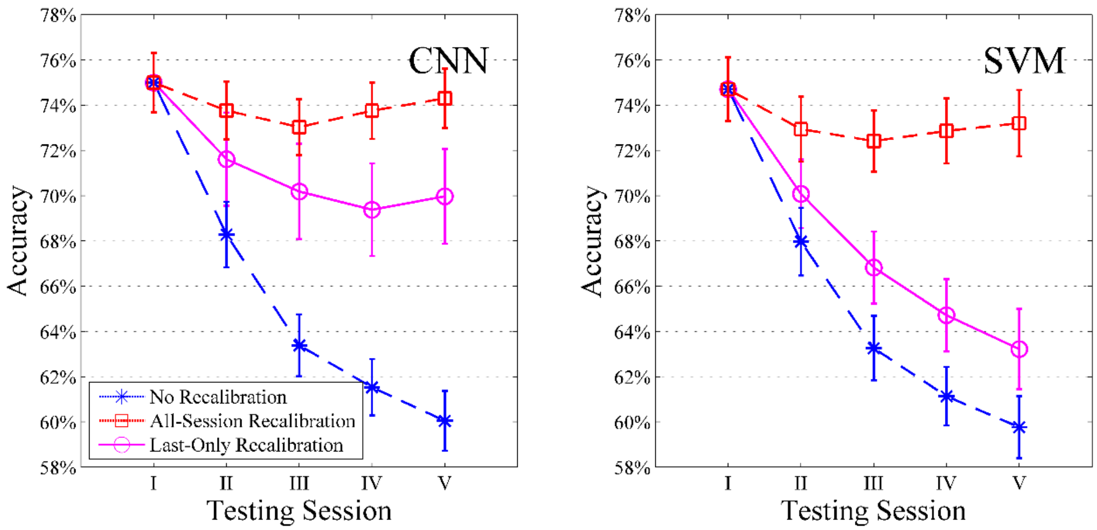

Supplementary Figure S6. Comparison of CNN and SVM in intact subjects (n=40) tested with 10 movement subset. Average session-to-session accuracy in different self-recalibration scenario. Repetition 1 of movement was used as the training data, and repetitions 2 to 6 were tested one by one with or without recalibration.

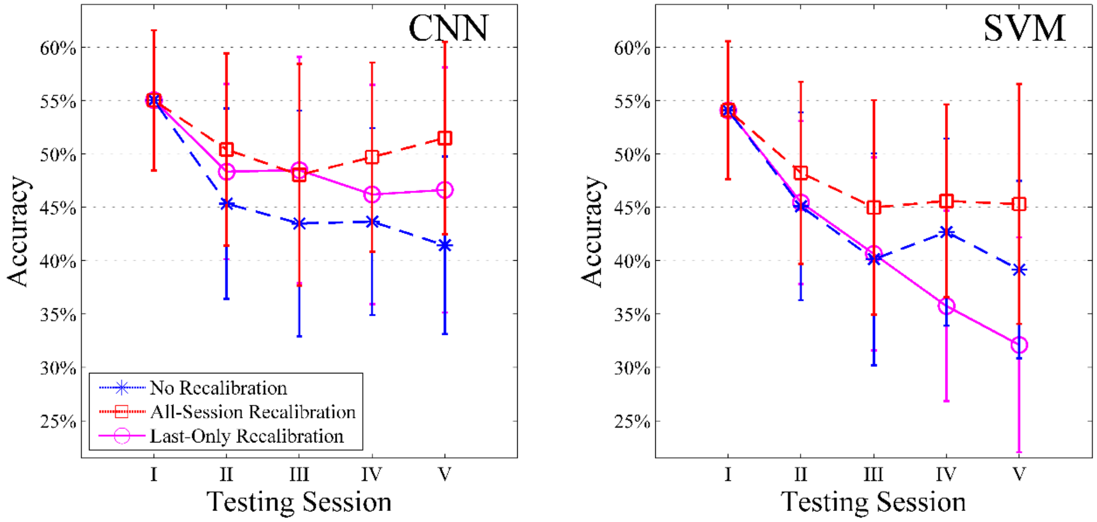

Supplementary Figure S7. Comparison of CNN and SVM in amputee subjects with remaining forearm >70% (n=4) tested with 10 movement subset. Average session-to-session accuracy in different self-recalibration scenario. Repetition 1 of movement was used as the training data, and repetitions 2 to 6 were tested one by one with or without recalibration.

Supplementary Table S1. Average movement-to- movement accuracy for baseline classifier and self-recalibration scenarios.

Index	Pattern Name	Baseline	Average accuracy over 5 testing sessions		
			No Recalibration	All Session	Last Only
1	Rest	61.61%	31.23%	35.34%	30.88%
2	Thumb up	93.28%	63.39%	72.72%	71.60%
3	Extension of index and middle, flexion of the others	86.76%	56.44%	69.63%	70.30%
4	Flexion of ring and little finger, extension of the others	83.13%	65.12%	70.40%	68.78%
5	Thumb opposing base of little finger	82.35%	48.71%	59.02%	59.43%
6	Abduction of all fingers	78.98%	54.85%	65.59%	65.56%
7	Fingers flexed together in fist	87.48%	66.09%	75.27%	74.41%
8	Pointing index	85.05%	63.03%	70.92%	67.69%
9	Adduction of extended fingers	80.43%	53.20%	59.76%	55.16%
10	Wist supination (axis: middle finger)	81.15%	46.56%	58.47%	59.80%
11	Wist pronation (axis: middle finger)	77.79%	51.48%	63.65%	63.23%
12	Wist supination (axis: little finger)	71.58%	37.70%	47.86%	45.54%
13	Wist pronation (axis: little finger)	77.35%	39.62%	52.40%	51.41%
14	Wrist flexion	82.98%	62.12%	72.23%	69.60%
15	Wrist extension	81.86%	61.55%	69.36%	67.44%
16	Wrist radial deviation	88.97%	57.69%	70.29%	67.24%
17	Wrist ulnar deviation	77.97%	48.26%	58.18%	59.44%
18	Wrist extension with closed hand	80.69%	66.64%	75.58%	71.52%
19	Large diameter grasp	70.52%	35.22%	44.02%	46.02%
20	Small diameter grasp (power grip)	66.63%	37.34%	47.59%	48.72%
21	Fixed hook grasp	69.09%	32.07%	39.71%	39.48%
22	Index finger extension grasp	79.41%	47.37%	61.47%	56.69%
23	Medium wrap	66.23%	39.55%	48.21%	41.52%
24	Ring grasp	74.19%	48.03%	61.30%	57.85%
25	Prismatic four fingers grasp	67.34%	30.33%	37.51%	36.52%
26	Stick grasp	72.98%	49.47%	60.81%	58.50%
27	Writing tripod grasp	66.31%	32.32%	40.20%	38.39%
28	Power sphere grasp	69.83%	40.08%	49.91%	47.17%
29	Three finger sphere grasp	66.12%	34.00%	44.17%	41.98%
30	Precision sphere grasp	60.99%	39.62%	48.29%	47.61%
31	Tripod grasp	66.41%	34.41%	40.79%	38.93%
32	Prismatic pinch grasp	68.13%	31.73%	37.69%	38.27%
33	Tip pinch grasp	72.07%	49.13%	56.77%	54.00%
34	Quadpod grasp	67.78%	33.82%	42.25%	40.83%
35	Lateral grasp	80.20%	53.71%	62.45%	61.03%
36	Parallel extension grasp	76.89%	46.66%	57.72%	55.61%
37	Extension type grasp	79.71%	52.55%	60.71%	60.10%
38	Power disk grasp	82.43%	60.39%	71.57%	69.57%
39	Open a bottle with a tripod grasp	76.89%	44.25%	57.18%	56.91%
40	Turn a screw (grasp the screwdriver with a stick grasp)	83.96%	57.74%	72.96%	69.87%
41	Cut something<br>(grasp the knife with an index finger extension grasp)	86.23%	67.34%	81.08%	80.30%
42	Flexion of the little finger	91.66%	62.74%	75.28%	74.20%
43	Flexion of the ring finger	89.08%	68.26%	78.14%	78.07%
44	Flexion of the middle finger	90.00%	70.26%	76.76%	75.51%
45	Flexion of the index finger	89.46%	70.17%	79.12%	77.15%
46	Abduction of the thumb	90.46%	76.76%	84.14%	83.31%
47	Flexion of the thumb	91.00%	70.27%	77.26%	77.30%
48	Flexion of index and little finger	88.57%	65.78%	75.62%	73.20%
49	Flexion of ring and middle finger	87.98%	78.61%	85.01%	84.23%
50	Flexion of index finger and thumb	87.63%	65.94%	72.41%	69.23%
